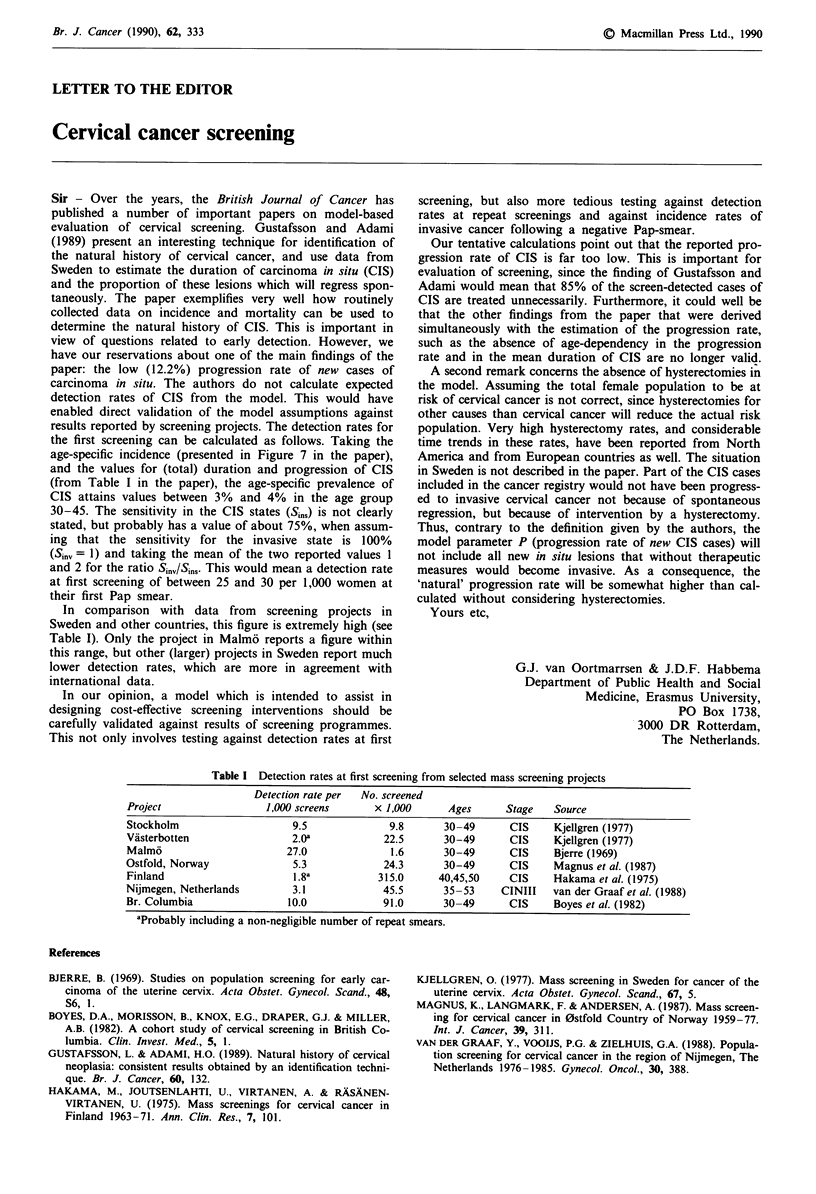# Cervical cancer screening.

**DOI:** 10.1038/bjc.1990.290

**Published:** 1990-08

**Authors:** G. J. van Oortmarrsen, J. D. Habbema


 © Macmillan Press Ltd., 1990

LETTER TO THE EDITOR

### Cervical cancer screening

**Sir** – Over the years, the *British Journal of Cancer* has published a number of important papers on model-based evaluation of cervical screening. Gustafsson and Adami (1989) present an interesting technique for identification of the natural history of cervical cancer, and use data from Sweden to estimate the duration of carcinoma *in situ* (CIS) and the proportion of these lesions which will regress spontaneously. The paper exemplifies very well how routinely collected data on incidence and mortality can be used to determine the natural history of CIS. This is important in view of questions related to early detection. However, we have our reservations about one of the main findings of the paper: the low (12.2%) progression rate of *new* cases of carcinoma *in situ*. The authors do not calculate expected detection rates of CIS from the model. This would have enabled direct validation of the model assumptions against results reported by screening projects. The detection rates for the first screening can be calculated as follows. Taking the age-specific incidence (presented in Figure 7 in the paper), and the values for (total) duration and progression of CIS (from Table I in the paper), the age-specific prevalence of CIS attains values between 3% and 4% in the age group 30–45. The sensitivity in the CIS states ($S_{ins}$) is not clearly stated, but probably has a value of about 75%, when assuming that the sensitivity for the invasive state is 100% ($S_{inv} = 1$) and taking the mean of the two reported values 1 and 2 for the ratio $S_{inv}/S_{ins}$. This would mean a detection rate at first screening of between 25 and 30 per 1,000 women at their first Pap smear.

In comparison with data from screening projects in Sweden and other countries, this figure is extremely high (see Table I). Only the project in Malmö reports a figure within this range, but other (larger) projects in Sweden report much lower detection rates, which are more in agreement with international data.

In our opinion, a model which is intended to assist in designing cost-effective screening interventions should be carefully validated against results of screening programmes. This not only involves testing against detection rates at first screening, but also more tedious testing against detection rates at repeat screenings and against incidence rates of invasive cancer following a negative Pap-smear.

Our tentative calculations point out that the reported progression rate of CIS is far too low. This is important for evaluation of screening, since the finding of Gustafsson and Adami would mean that 85% of the screen-detected cases of CIS are treated unnecessarily. Furthermore, it could well be that the other findings from the paper that were derived simultaneously with the estimation of the progression rate, such as the absence of age-dependency in the progression rate and in the mean duration of CIS are no longer valid.

A second remark concerns the absence of hysterectomies in the model. Assuming the total female population to be at risk of cervical cancer is not correct, since hysterectomies for other causes than cervical cancer will reduce the actual risk population. Very high hysterectomy rates, and considerable time trends in these rates, have been reported from North America and from European countries as well. The situation in Sweden is not described in the paper. Part of the CIS cases included in the cancer registry would not have been progressed to invasive cervical cancer not because of spontaneous regression, but because of intervention by a hysterectomy. Thus, contrary to the definition given by the authors, the model parameter *P* (progression rate of *new* CIS cases) will not include all new *in situ* lesions that without therapeutic measures would become invasive. As a consequence, the 'natural' progression rate will be somewhat higher than calculated without considering hysterectomies.

Yours etc,

G.J. van Oortmarrsen & J.D.F. Habbema
Department of Public Health and Social Medicine, Erasmus University,
PO Box 1738,
3000 DR Rotterdam,
The Netherlands.

**Table I** Detection rates at first screening from selected mass screening projects

| *Project* | *Detection rate per 1,000 screens* | *No. screened × 1,000* | *Ages* | *Stage* | *Source* |
|---|---|---|---|---|---|
| Stockholm | 9.5 | 9.8 | 30–49 | CIS | Kjellgren (1977) |
| Västerbotten | 2.0[a] | 22.5 | 30–49 | CIS | Kjellgren (1977) |
| Malmö | 27.0 | 1.6 | 30–49 | CIS | Bjerre (1969) |
| Ostfold, Norway | 5.3 | 24.3 | 30–49 | CIS | Magnus *et al.* (1987) |
| Finland | 1.8[a] | 315.0 | 40,45,50 | CIS | Hakama *et al.* (1975) |
| Nijmegen, Netherlands | 3.1 | 45.5 | 35–53 | CINIII | van der Graaf *et al.* (1988) |
| Br. Columbia | 10.0 | 91.0 | 30–49 | CIS | Boyes *et al.* (1982) |

[a]Probably including a non-negligible number of repeat smears.

**References**

BJERRE, B. (1969). Studies on population screening for early carcinoma of the uterine cervix. *Acta Obstet. Gynecol. Scand.*, **48**, S6, 1.

BOYES, D.A., MORISSON, B., KNOX, E.G., DRAPER, G.J. & MILLER, A.B. (1982). A cohort study of cervical screening in British Columbia. *Clin. Invest. Med.*, **5**, 1.

GUSTAFSSON, L. & ADAMI, H.O. (1989). Natural history of cervical neoplasia: consistent results obtained by an identification technique. *Br. J. Cancer*, **60**, 132.

HAKAMA, M., JOUTSENLAHTI, U., VIRTANEN, A. & RÄSÄNEN-VIRTANEN, U. (1975). Mass screenings for cervical cancer in Finland 1963–71. *Ann. Clin. Res.*, **7**, 101.

KJELLGREN, O. (1977). Mass screening in Sweden for cancer of the uterine cervix. *Acta Obstet. Gynecol. Scand.*, **67**, 5.

MAGNUS, K., LANGMARK, F. & ANDERSEN, A. (1987). Mass screening for cervical cancer in Østfold Country of Norway 1959–77. *Int. J. Cancer*, **39**, 311.

VAN DER GRAAF, Y., VOOIJS, P.G. & ZIELHUIS, G.A. (1988). Population screening for cervical cancer in the region of Nijmegen, The Netherlands 1976–1985. *Gynecol. Oncol.*, **30**, 388.